# Morphological study of larynx, trachea, and lungs of *Didelphis marsupialis* (LINNAEUS, 1758)

**DOI:** 10.14202/vetworld.2020.2142-2149

**Published:** 2020-10-13

**Authors:** Bruna Tassia Santos Pantoja, Armando Reinaldo Marques Silva, Renata Mondego-Oliveira, Thamires Santos Silva, Babara Carvalho Marques, Rafaela Pontes Albuquerque, Joicy Cortez Sá Sousa, Rose Eli Grassi Rici, Maria Angélica Miglino, Alana Lislea Sousa, André Luís Resende Franciolli, Eduardo Martins Sousa, Ana Lúcia Abreu-Silva, Rafael Cardoso Carvalho

**Affiliations:** 1 Graduate Program in Anatomy of Domestic and Wild Animals, Department of Surgery, Faculty of Veterinary Medicine and Animal Science, University of São Paulo, São Paulo, Brazil; 2 Center for Agricultural and Environmental Sciences, Federal University of Maranhão, Chapadinha, Brazil; 3 Northeast Biotechnology Network, Center for Biological and Health Sciences, Federal University of Maranhão, São Luís, Brazil; 4 Graduate Program in Health Sciences, Center for Biological and Health Sciences, Federal University of Maranhão, São Luís. Brazil; 5 Graduate Program in Microbial Biology, CEUMA University, São Luís, Brazil; 6 Graduate Program in Animal Science, Faculty of Veterinary Medicine, Center for Agricultural Sciences, University of Maranhão, São Luís, Brazil

**Keywords:** anatomy, histology, marsupials, respiratory tract

## Abstract

**Background and Aim:**

From a biomedical point of view, the value of marsupials as a model of primitive mammals is indisputable. Among its species, the possum is a model that allows the study of the ontogeny of different organic systems, as well as their physiological aspects. The relevance of anatomical, functional, evolutionary, and phylogenetic study of marsupials for the development of comparative morphology is extensively documented in the literature. However, there are still many aspects to be further evaluated, as the anatomy and histology of the respiratory tract of this species. The aim of this study was to describe the morphology of the larynx, trachea, and lungs of *Didelphis marsupialis*.

**Materials and Methods:**

Five adult male animals were donated to the Comparative Animal Anatomy Laboratory – LAAC/CCAA-UFMA, for morphological studies. Specimens were washed in running water to perform biometrics. Then, they were fixed with 10% formaldehyde solution. After the fixation period, the specimens were positioned in dorsal decubitus position, for dissection of the respiratory system organs, by opening the ventral region of the neck and thoracic cavity, with subsequent removal of the pectoral muscles, ribs, and sternum. For histological analysis, fragments of 1 cm^2^ of the larynx (epiglottis and thyroid cartilages), trachea, and lungs were collected and fixed in 10% formaldehyde solution. Right after fixation, the fragments were dehydrated in increasing concentrations of ethyl alcohol (70, 80, 95, and 100%), diaphanized in xylene, embedded in paraffin, and sectioned into thin slices of 5 μm using a microtome. Sections were stained using the hematoxylin and eosin technique.

**Results:**

Anatomically, the larynx starts right after the pharynx. It consisted of four cartilages: Epiglottis, cricoid, thyroid, and arytenoid. The trachea was made of dorsally incomplete cartilaginous rings. At the entrance of the thoracic cavity, it bifurcated into the left and right main bronchus. The left lung was smaller than the right lung, with two lobes (cranial and caudal). The right lung presents the cranial, middle, caudal, and accessory lobes. Histologically, the epiglottis consisted of elastic cartilage and is covered by a non-keratinized stratified squamous epithelium. Thyroid cartilage is made of hyaline cartilage covered by smooth muscle. The trachea presents hyaline cartilage, with ciliated pseudo-stratified epithelium, serous glands, isogenic groups of chondrocytes, and perichondrium. The lung consisted of bronchi, bronchioles, and alveoli, also presenting blood vessels and arteries.

**Conclusion:**

Morphologically, the larynx, trachea, and lungs of *D. marsupialis* were similar to those of the other *Didelphids* described in the literature.

## Introduction

Marsupials, as primitive mammal models, have undeniable biomedical value. Experimental research related to capture, feeding, captive maintenance, anesthetic protocols [1], collection of body fluids [2,3], and perfusion techniques for fixing samples for histology [4] is frequent. In addition, the relevance of anatomical, functional, evolutionary, and phylogenetic study of marsupials for the development of comparative morphology is extensively documented [5].

The wide geographical distribution of *Didelphids marsupialis* means that these individuals are represented in most of the studies on the ecology of communities and populations [6]. They are animals that have varied eating habits and habitats [7], found in virgin forest, cultivated areas, growing vegetation, and urban areas [8]. Among the *Didelphids*, the genus *Didelphis* is distributed between North and South America. In South America, the species are divided into two groups: White-eared opossums (*Didelphis albiventris* and *Dentinogenesis imperfecta*) and black-eared opossums (*Didelphis aurita* and *Didelphis marsupialis*), the latter two occupying forest areas [9].

*D. marsupialis* is a marsupial commonly found in the Amazon region and on the banks of Cerrado. It is popularly known as “*mucura*” and opossum. They are nocturnal mammals [7,10], seeking shelter in tree hollows, between roots and houses liner [11,12]. It has general food habits, with varied diet from animal sources such as insects, small vertebrates and birds, and plant sources, leaves, and fruits [6]. The external morphology is characterized by a gray or black dorsal color; the presence of long white hairs, which stand out from black hairs; by the rough texture of the coat. The ventral color is yellowish cream. Facial coloring is usually black or gray, with no conspicuous marks on the face. The ears are large, dark, and hairless [13]. It has sexual dimorphism, observed in relation to the width and length of the skull (females have the narrowest head). Males, on the other hand, show more accelerated growth, as they do not present energy costs with pregnancy and lactation [14].

The need for preservation and protection of wild species also demands greater knowledge about their morphology [15]. *D. marsupialis* has great biological importance but, despite this, several aspects still need to be studied more deeply, such as, for example, the morphological aspects of the respiratory system. The data in the classical literature regarding the morphology of this system are non-existent and, even in specific studies, the findings are scarce, some of them inaccurate, which justifies this study.

In this context the aim of this study was to describe morphologically larynx, trachea, and lungs of *Didelphis marsupialis*, in order to provide data that can collaborate with anatomical, biological and scientific knowledge about this wild species of Brazilian fauna.

## Materials and Methods

### Ethical approval

This research obtained authorization from the Ethics Committee on the Use of Animals (CEUA/UFMA – protocol nº 23115.005452/2016-61) and SISBIO – ICMBio (protocol nº 58272-1). All protocols for the use of animals in this research are in accordance with the legislation imposed by the National Council for the Control of Animal Experimentation (CONCEA), Brazil.

### Study period and location

The study was performed from August 2018 to December 2019 at the Comparative Animal Anatomy Laboratory, Center for Agricultural and Environmental Sciences at the Federal University of Maranhão - Chapadinha, Maranhão, Brazil (3 ° 44 ′ 31 ″ S, 43 ° 21 ′ 36 ″ W).

### Animals, macroscopic and microscopic analysis

Five male adult animals were used for this research. Since they present a synanthropic behavior, they are commonly found dead around car traffic areas. In such cases, they were donated to the Comparative Animal Anatomy Laboratory – LAAC/CCAA-UFMA by the people living nearby the Federal University of Maranhão. After receiving the animals, they were washed in running water and fixed with a 10% formaldehyde solution. The solution was injected into the musculature, chest, abdomen, as well as the body cavities of each animal, using a 40×12 needle and a syringe. Then, specimens were immersed in a container with 10% formaldehyde for 48 h. After the fixation period, the specimens were washed in running water and positioned in dorsal decubitus position, for dissection of the organs of respiratory system. An incision was made in the midline, starting in the ventral region of the neck to the pelvic symphysis, with subsequent opening of the ventral region of the neck, to access the ventral cervical region, followed by the ventral opening of the thoracic cavity, with subsequent removal of the pectoral muscles, ribs, and sternum, to access the organs of the thoracic cavity.

For histological analysis, fragments of 1 cm^2^ of the epiglottis, thyroid, cricoid and arytenoid cartilages, trachea, and lungs were collected and fixed with 10% formaldehyde solution. Right after fixation, the fragments were dehydrated in increasing concentrations of ethyl alcohol (70, 80, 95, and 100%), diaphanized in xylene, embedded in paraffin, and sectioned into thin slices of 5 μm using a microtome. Sections were stained using the hematoxylin and eosin technique [16]. After each analysis, all the samples were photodocumented.

## Results

In *D. marsupialis*, the larynx was in the ventral part of the neck, rostral to the trachea. It was a short tubular organ, which connects the pharynx to the trachea, relatively short and wide, located in a superficial position, ventrally to the first and second cervical vertebrae. It is dorsally related to the pharynx and the esophagus, laterally to the sternothyroid muscle and to the mandibular salivary gland and ventrally to the sternohyoid muscle.

**Figure-1:**
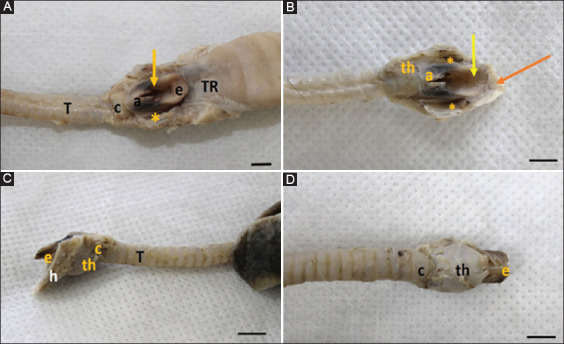
Larynx and trachea of *Didelphis marsupialis* “*ex situ*.” Legend: (A) and (B) – larynx of *D. marsupialis* “*ex situ*,” in dorsal view, where the larynx is related rostrally to the tongue root (TR) and caudally to the trachea (T). Note the epiglottis (e), with an oval shape and rounded apex. Yellow arrow – laryngeal aditus; * – piriform recess; a – arytenoid; c – cricoid cartilage. (b) – observe the thyroid cartilage (th) and the hyoid bone (orange arrow). Bar: 0.5 cm. (C) – larynx of *D. marsupialis* “*ex situ*,” in the left lateral view. Observe the laryngeal cartilages and the hyoid bone, as the bone base of the tongue and larynx and e – epiglottis; h – hyoid bone; th – shield-shaped thyroid cartilage; c – cricoid cartilage. Bar: 0.5 cm. (D) – larynx of *D. marsupialis* “*ex situ*,” in ventral view. Observe the thyroid cartilages (th), with the shape of a shield, with evidenced laryngeal prominence; in c – the U-shaped cricoid cartilage; and e – epiglottis (apex). Bar: 0.5 m.

**Figure-2:**
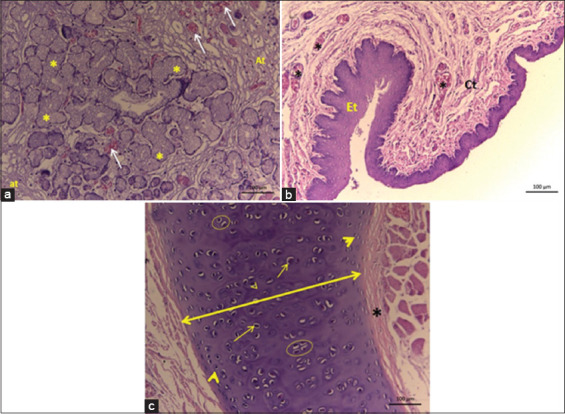
Histology of the epiglottis cartilage of *Didelphis marsupialis*. Legends: (a) – Mixed acids (*), blood vessels (arrows), and adipose tissue (Ta); (b) – non-keratinized stratified squamous epithelial tissue (Et), connective tissue (Ct), and blood vessels (*); (c) – elastic cartilage (double arrow), perichondrium (*), isogenic groups of chondrocytes (circle), chondrocytes (arrow), and chondroblasts (arrowhead).

It consisted of four hyaline and elastic cartilages: Thyroid, epiglottis, arytenoid, and cricoid (Figures-1A-1D). The epiglottis cartilage had an oval shape, with a rounded apex. The tongue root was located caudally and rostrally to the thyroid and arytenoid cartilages. This cartilage leans dorsorostrally behind the soft palate and its anatomical disposition leads us to infer that it could lean in a retrograde movement to partially cover the entrance to the larynx at the time of swallowing. It consisted of elastic cartilage surrounded by the perichondrium (dense connective tissue) (Figure-2). It was covered by non-keratinized stratified squamous epithelium (Figure-2).

**Figure-3:**
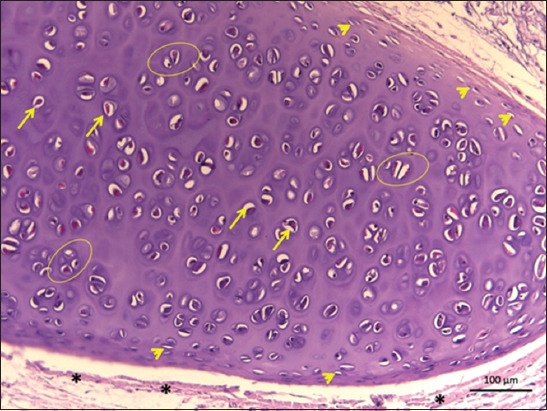
Histology of the thyroid cartilage of *Didelphis marsupialis*. Legend: Chondroblasts (arrowhead), chondrocytes (arrows), isogenic groups (circle), muscle tissue (*). Hyaline type cartilage.

The thyroid cartilage had a shield shape and was the largest of the laryngeal cartilages. It was located rostrally to the cricoid cartilage, being divided into two cartilage plates (right and left), which are fused ventrally. Its inner part was concave, with a smooth appearance, and the outer part is convex, with few apparent roughness. It consisted of hyaline-type cartilage, with isogenic groups, and was covered by smooth muscle (Figure-3).

The cricoid cartilage had a “U” shape, located rostrally to the trachea and caudally to the thyroid cartilage. The arytenoid cartilage had a shell shape. In our study, the corniculate process of the arytenoid cartilage was not observed. It was important to note that in this species, the laryngeal cartilages were covered by extrinsic muscles, which allowed their movement.

**Figure-4:**
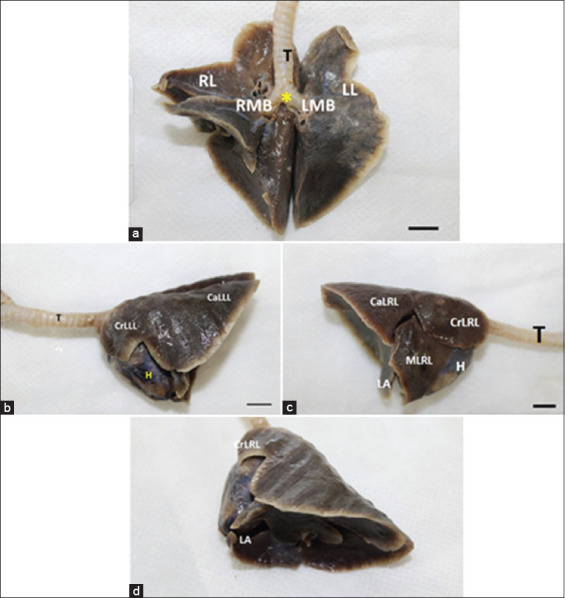
Trachea, main bronchi, and lungs of *Didelphis marsupialis* “*ex situ*.” Legend: (a) – trachea, left and right main bronchi of *D. marsupialis* “*ex situ*,” in ventral view. Observe the pattern of bronchial tree formation, with the bifurcation of the trachea (T) in the right main bronchus (RMB) and left main bronchus (LMB) with the tracheal carina (*) in evidence. Right lung (RL) and left lung (LL) and their lobes. Bar: 1 cm. (b) – Left lung in evidence (left side view), where the cranial lobe of the left lung (CrLLL) and the caudal lobe of the left lung (CaLLL) can be seen, although the presence of the interlobar fissure, which would separate these lobes, is not evident; (c) – heart. Bar: 1 cm. (c) – Right lung in evidence (right side view). Note the cranial lobes of the right lung (CrLRL), the middle lobe of the right lung (MLRL), the caudal lobe of the right lung (CaLRL), and part of the accessory lobe (AL); (c) – heart. Note that the right lung has more lobes and anatomically, a larger volume than the left lung. Bar: 1 cm. (d) – Lung in diaphragmatic view. Note the AL and its topographic relationship with the heart (H); in this anatomical view, it can also be observed the costal impressions on the lateral surface of the left lung, and the diaphragmatic impression on the diaphragmatic surface of the left and right lungs; CLLL – Cranial lobe of the left lung. Bar: 1 cm.

**Figure-5:**
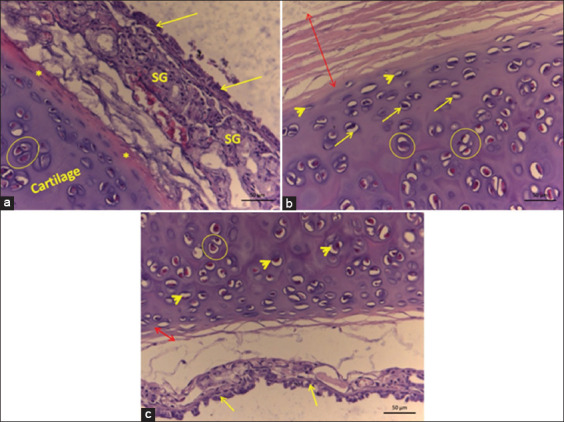
Histology of the trachea of *Didelphis marsupialis*. Legend: (a) – Ciliary pseudostratified epithelium (arrow), perichondrium (*), chondrocyte isogenic groups (circle), serous glands (SG); (b) – perichondrium (double arrow), chondroblasts (arrowhead), chondrocytes (arrow), chondrocyte isogenic groups (circle); (c) - hyaline type cartilage, note ciliated pseudostratified epithelium (arrow), chondrocytes (arrowhead), chondrocyte isogenic groups (circle), and perichondrium (double arrow).

The trachea (Figures-1c and d, 4a-c) of *D. marsupialis* presents itself as a cartilaginous, flexible, and semi-rigid tube, which connects the larynx to the main bronchi. It consisted of tracheal rings, joined ventrally and laterally by the annular ligaments and dorsally by the tracheal dorsal membrane, so they were incomplete dorsally. Anatomically, the trachea had a cervical and a thoracic portion, according to the body region in which it was located. After the last tracheal ring, in the region of the pulmonary hilum, the trachea bifurcated to form the tracheal carina (Figure-4a), which originated two short pulmonary bronchi (left and right), called primary bronchi (Figure-4a and b). Histologically, it had a ciliated pseudostratified epithelium (Figure-5c), with goblet cells, serous glands (Figure-5a), chondrocyte isogenic groups (Figure-5a and c), and perichondrium (Figure-5b). Its epithelium was characteristic of the respiratory tract, where goblet cells were located, responsible for mucus secretion.

**Figure-6:**
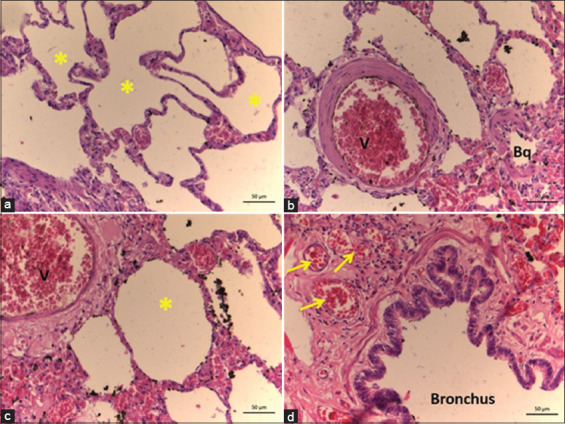
Histology of the lung of *Didelphis marsupialis*. Legend: (a) – Alveoli (*); (b) – blood vessel (V – artery), bronchioles (Bq); (c) – blood vessel (V – artery), alveolus (*); (d) – bronchus, vein (arrow), connective tissue (double arrow).

The lungs of *D. marsupialis* were located inside the thoracic cavity, between the first and tenth ribs. In this species, each lung was invaginated in the pleural sac, covered by the pulmonary pleura and moving freely in that area. These organs were attached only by their root and by the pulmonary ligament. The lungs have deep interlobar fissures, which divided the organ into lobes. Anatomically, the right lung had four lobes (cranial, caudal, middle, and accessory) and the left lung was divided into two lobes, cranial and caudal, with no apparent lobation divisions (Figures-4b-d). In the species studied, the right lung was almost twice the size of the left lung, as it had an accessory lobe and the cranial lobe was larger than that of the left lung (“*in situ*” observation). In this species, the right lung extended to the left side of the thoracic cavity, dorsally to the trachea and to the great vessels, being located cranioventrally to the heart, in the pericardial sac. Histologically, the pulmonary lobes were covered by a thin layer of connective tissue. The pulmonary parenchyma consisted of bronchi, which taper to form the bronchioles. The inner lining was formed by simple cuboidal epithelium, covered by a smooth muscle ring (Figure-6). The alveolar sacs were clustered, in the form of rosettes, dispersed in a thin layer of loose connective tissue, blood vessels, and smooth muscle. In general, as the conductive airways were subdivided, they became progressively smaller in diameter. The respiratory epithelium became shorter, with fewer goblet cells. The number of glands, connective tissue, and cartilage also decreased.

## Discussion

The opossum is a wild animal of great ecological importance, as an effective seed disperser [17]. However, there are still few studies on the anatomy of this animal. In the literature, anatomical descriptions of the structures of the respiratory system are scarce and, when referring to the species in question, the data are non-existent or, when present, described in general terms, based on the analogy of close species or general characteristics of the species of Didelphidae family.

The respiratory system has the function of promoting gas exchange between the internal and external environment of the organism: It supplies oxygen to the different tissues of the body, along with the circulatory system, and eliminates carbon dioxide produced in those same tissues [18]. In mammals, the respiratory system is formed by the airways, represented by the nostrils, nasal cavity, pharynx, larynx, trachea, bronchi, and bronchioles and by the lungs, where gas exchange between blood and atmospheric air occurs [19]. The respiratory system of mammals, in general, consists of a conductive portion, a respiratory portion, and a pumping apparatus [20].

The larynx is a musculocartilaginous organ which connects the pharynx to the trachea. It acts on phonation and prevents the aspiration of foreign material [19]. The knowledge of the comparative morphology of the larynx among wild species is important and supports evolutionary and functional discussions about its role in breathing and vocalization [21,22]. As for the general characteristics of laryngeal cartilages, our descriptions are similar to domestic species [20,23]. However, when comparing our results with those of other species of Didelphidae family, we note some striking anatomical differences. A study with *Didelphis* spp. opossums showed that the cricoid cartilage has a “V” shape. In addition, the authors report the presence of the elastic corniculate process in the arytenoid cartilage [24], differently from what was observed in our observation. These same authors carried out the histological study of these cartilages and observed that they are predominantly hyaline, similar to our findings. By analogy, it can be assumed that these cartilages are susceptible to changes with age, such as ossification, for example, as in primates [25].

In *Nasua nasua*, the laryngeal cartilages are covered by extrinsic muscles, which allow them to move [26]. In domestic carnivores, the larynx is situated in a superficial position, ventrally to the first and second cervical vertebrae, dorsally related to the pharynx and esophagus, laterally to the sternothyroid muscle and to the mandibular salivary gland, and ventrally to the sternohyoid muscle [20,23], similar to that observed in *D. marsupialis*.

In *D. marsupialis*, the epiglottis cartilage has an oval shape, with a rounded apex, different from that described for *D. albiventris*, which has leaf-shaped cartilage with a pointed apex [24]. This difference can be attributed to the anatomical peculiarity inherent to the different species within the same family [27]. Histologically, the epiglottis in the species studied has the same characteristics as observed in other mammals such as elastic cartilage surrounded by perichondrium type (dense connective tissue) lined by non-keratinized stratified squamous epithelial tissue [28,29].

The trachea of mammals, reptiles, and amphibians is a flexible, semi-rigid cartilaginous tube that connects the larynx to the main bronchi, composed of tracheal rings joined ventrally and laterally by the annular ligaments and dorsally by the tracheal dorsal membrane [27]. In *Myocastor coypus*, the trachea is described as an organ formed by hyaline cartilage and a lining epithelium characteristic of the respiratory tract, where goblet cells, responsible for mucin secretion, are located [30]. It is important to highlight that these characteristics can also be attributed to *D. marsupialis*.

In dogs [31], cats [20], and coatis [31,32], after the last tracheal ring, in the region of the pulmonary hilum, the trachea bifurcates to form the tracheal carina, which originates two short pulmonary bronchi (left and right), called primary bronchi. Then, the primary bronchi send branches for each of the four right lobes (cranial, caudal, middle, and accessory) and left lobes (cranial and caudal), called secondary or lobar bronchi. This was also observed in our findings.

The lungs are located within the chest cavity, between the first and tenth ribs. Each lung is surrounded by the pleura and moves freely within it. These organs were attached only by the root and the pulmonary ligament. They have deep interlobar fissures, which divide the organ into lobes. These characteristics are present in domestic carnivores [20], *N. nasua* [26,32,33], *Mazama gouazoubira* [34], *Procyon cancrivorus* [35], and *D. marsupialis*. The interlobar fissures allow a better adaptation of the lungs to changes resulting from respiratory movements [20]. There is also the statement that these fissures are not used as systematic evidence [27], although they demonstrate adaptive variations, due to the different numbers of pulmonary lobes and fissures found in different species [36,37].

Regarding pulmonary lobation, the classic veterinary literature clarifies the following pattern: Right lung with four lobes: Cranial, caudal, medium, and accessory and left lung divided into two lobes, cranial and caudal [19,20,23]. The results observed in our research are similar to those found in other mammals: Carnivores, such as dogs [31], *Leopardus pardalis* [38], *N. nassua* [32,33], and *P. cancrivorus* [35]; rodents, such as *Hydrochoerus hydrochaeris* [39]; *Xenarthra*, such as *Myrmecophaga tridactyla* [40]; and primates, such as *Callithrix penicillata* [41]. Although there is great variation in macroscopic and pulmonary lobation characteristics in several species of wild mammals, only phylogenetic studies will be able to show whether there is a pattern for bronchial lobation in different mammalian orders [27].

## Conclusion

Morphologically, the organs of the respiratory system follow the same pattern already described for carnivores, rodents, and other marsupials, which serve as further evidence of the importance of *D. marsupialis* as a study model.

## Authors’ Contributions

BTSP, ARMS, TSS, and BCM carried out anatomical analysis. RMO, RPA, JCSS, REGR, and ALRF performed histological analysis. ALS, EMS, and MAM provided reagents for histological techniques and supervised and revised the manuscript. ALAS carried out data analysis, manuscript preparation, revision, and submission of the manuscript. RCC designed the study, analyzed data, revised the manuscript, and contributed to the study. All authors have read and approved the final manuscript.
